# Is faster better? Operative duration in emergent appendicectomy

**DOI:** 10.1186/1753-6561-9-S7-A29

**Published:** 2015-10-27

**Authors:** RT D'cruz, E McDermott

**Affiliations:** 1 School of Medicine and Medical Sciences, University College Dublin, Dublin, Republic of Ireland; 2 St. Vincent's University Hospital, Dublin, Republic of Ireland

## Background

For many years, acute appendicitis has been regarded as a condition that required urgent surgical treatment. Emergent appendicectomy has been accepted generally as the most appropriate treatment, despite the lack of objective proof. The timing of appendicectomy has been investigated in the adult population but no definite conclusion has been made.

## Purpose

The objective of this study was to investigate the effect of operative duration of appendicectomy on outcomes such as complications and length of stay.

## Methods

A retrospective study of 271 patients who had undergone appendicectomy from 1 January 2013 to 31 December 2013 was conducted. Data that includes time of presentation to Emergency Department, presenting complaint, diagnostic investigations, time of operation, length of hospital stay and complications were collected. These patients were subsequently put into categories based on the duration of appendicectomy i.e. <45 mins, 46 - 60 mins, 61 - 75 mins, 76 - 90 mins & >90 mins.

## Results

182 patients had at least one form of diagnostic imaging modality performed. Pre-operative laboratory investigations were conducted in most patients, that included full blood count(FBC), Urea & Electrolytes (U&E) and inflammatory marker CRP. 193 out of 261 patients (73.9%) had their surgery within 24 hours of presentation. 253 patients (93.3%) had undergone laparoscopic appendicectomy. The average operating time was 63.8 mins. There was an exponential correlation between the operative duration and the length of hospital stay. There was no significant difference in the incidence of complications of acute appendicitis.

## Conclusions

The timing of appendicectomy was associated with increased length of stay. The optimal timing of completion of appendicectomy for acute appendicitis would be within 60 mins from the time of skin incision. However, operative duration did not affect the incidence of complications of acute appendicitis.
